# Crystal structure of THEP1 from the hyperthermophile *Aquifex aeolicus*: a variation of the RecA fold

**DOI:** 10.1186/1472-6807-5-7

**Published:** 2005-03-20

**Authors:** Michael Roßbach, Oliver Daumke, Claudia Klinger, Alfred Wittinghofer, Michael Kaufmann

**Affiliations:** 1 Institute of Neurobiochemistry, The Protein Chemistry Group, Witten/Herdecke University, Stockumer Strasse 10, 58448 Witten, Germany; 2 Max Planck Institute of Molecular Physiology, Department of Structural Biology, Otto-Hahn-Strasse 11, 44227 Dortmund, Germany

## Abstract

**Background:**

aaTHEP1, the gene product of *aq_1292 *from *Aquifex aeolicus*, shows sequence homology to proteins from most thermophiles, hyperthermophiles, and higher organisms such as man, mouse, and fly. In contrast, there are almost no homologous proteins in mesophilic unicellular microorganisms. aaTHEP1 is a thermophilic enzyme exhibiting both ATPase and GTPase activity *in vitro*. Although annotated as a nucleotide kinase, such an activity could not be confirmed for aaTHEP1 experimentally and the *in vivo *function of aaTHEP1 is still unknown.

**Results:**

Here we report the crystal structure of selenomethionine substituted nucleotide-free aaTHEP1 at 1.4 Å resolution using a multiple anomalous dispersion phasing protocol. The protein is composed of a single domain that belongs to the family of 3-layer (α/β/α)-structures consisting of nine central strands flanked by six helices. The closest structural homologue as determined by DALI is the RecA family. In contrast to the latter proteins, aaTHEP1 possesses an extension of the β-sheet consisting of four additional β-strands.

**Conclusion:**

We conclude that the structure of aaTHEP1 represents a variation of the RecA fold. Although the catalytic function of aaTHEP1 remains unclear, structural details indicate that it does not belong to the group of GTPases, kinases or adenosyltransferases. A mainly positive electrostatic surface indicates that aaTHEP1 might be a DNA/RNA modifying enzyme. The resolved structure of aaTHEP1 can serve as paradigm for the complete THEP1 family.

## Background

Comparative genomics led to the definition of 4873 clusters of orthologous groups of proteins (COGs) by comparing protein sequences encoded in (currently 66) completely sequenced genomes [1]. Aimed at finding thermophile-specific proteins among bacteria, extended phylogenetic patterns searches based on the COG-database were performed. Using this strategy, COG1618 was detected as a cluster containing proteins from all thermophilic and hyperthermophilic but only one mesophilic organism [2-4]. Surprisingly, although also absent from unicellular eukaryotes, COG1618-homologs are present in many higher multicellular organism such as *Homo sapiens*, *Mus musculus*, *Danio rerio*, *Rattus norvegicus*, etc. Because of this unusual phylogenetic distribution, aaTHEP1, the gene product of *aq_1292 *from the hyperthermophilic bacterium *Aquifex aeolicus*, was characterised biochemically as the first member of COG1618 proteins [2]. The analysis revealed that aaTHEP1 is an NTPase catalyzing ATP and GTP hydrolysis at turnover rates of 5 × 10^-3 ^s^-1^and 9 × 10^-3 ^s^-1^, respectively, with a K_m _in the micromolar range and a temperature optimum between 70 and 80°C. Although COG1618 proteins are annotated as "predicted nucleotide kinases"such an activity could not be confirmed for aaTHEP1 experimentally and its *in vivo *function remains unknown. To further characterize the aaTHEP1 function, we resolved its three dimensional structure by X-ray crystallography.

## Results and discussion

### Overall structure, domain class and architecture

Selenomethionine substituted aaTHEP1 was purified as described earlier [2] and eluted as a monomer from the final gel filtration column. Analysis of its nucleotide loading state using HPLC revealed that it was partially loaded with ADP (approx. 30%, data not shown). It was crystallized using PEG3350 as precipitant in the presence of KH_2_PO_4 _(see Methods) and crystals diffracted up to 1.4 Å using synchrotron radiation (see Table 1). Initial phases were obtained using a MAD phasing protocol (see Methods) and a model was build and refined. The final model has an R_cryst _of 16.8% and an R_free _of 20.8% and contains one aaTHEP1 molecule in the asymmetric unit. 172 amino acid residues, 249 water molecules, one phosphate, one magnesium and two sodium ions were included in the model. No electron density was found for residues D38-K43 which are part of a disordered loop.

**Figure 1:**
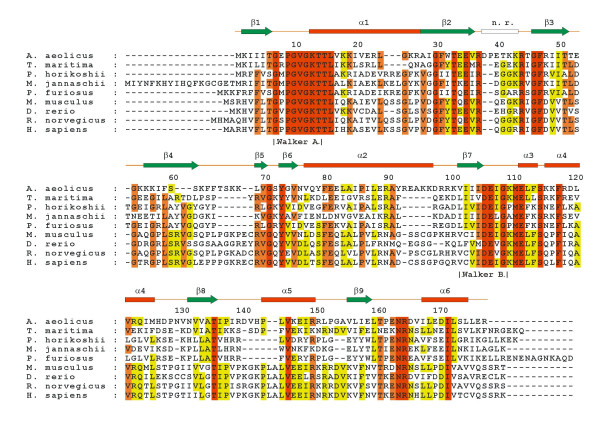
**Secondary structure of aaTHEP1 and multiple sequence alignment to homologous sequences**. Multiple sequence alignment of aaTHEP1 with the four most homologous sequences from both, thermophiles and eukaryotes in the order as they were detected by BLAST. The aligned sequences are THEP1 from *Aquifex aeolicus *VF5 (aaTHEP1, accession number NP_213886), THEP1 from *Thermotoga maritima *MSB8 (tmTHEP1, accession number NP_227852), THEP1 from *Pyrococcus horikoshii *OT3 (phTHEP1, accession number NP_142728), THEP1 from Methanocaldococcus jannaschii DSM 2661, accession number NP_248567), THEP1 from *Pyrococcus furiosus *DSM 3638 (accession number NP_578230), THEP1 from *Mus musculus *(mmTHEP1, accession number NP_079912), THEP1 from *Danio rerio *(drTHEP1, accession number NP_001003463), THEP1 from *Rattus norvegicus *(rnTHEP1, accession number XP_341723) and THEP1 from *Homo sapiens *(hsTHEP1, accession number NP_115700). The secondary structure of aaTHEP1 as calculated by DSSP and the locations of the Walker A (P-loop) and Walker B motif are shown with reference to the aaTHEP1 sequence. Six amino acids that could not be resolved are indicated by a white box (n. r.). Conservation of residues by 100%, 80% and 60% are coloured red, orange and yellow, respectively.

**Figure 2:**
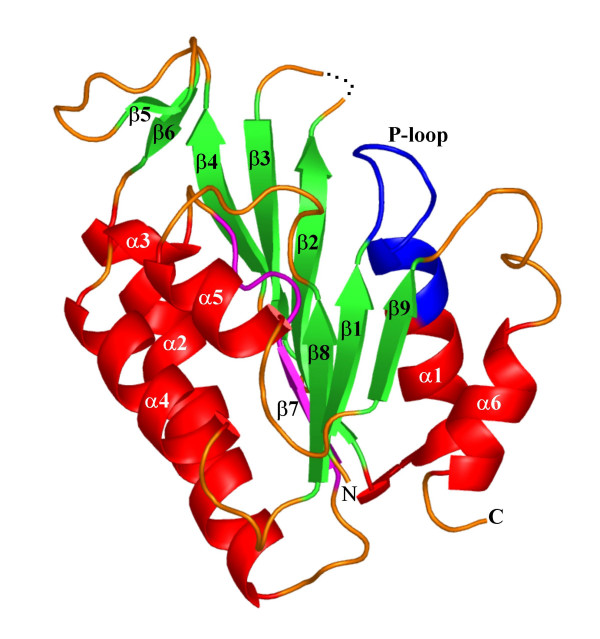
**Three dimensional structure of aaTHEP1**. Ribbon representation of the overall three dimensional structure of aaTHEP1. Walker A (P-loop) and Walker B motifs are coloured in blue and magenta, respectively.

**Figure 3:**
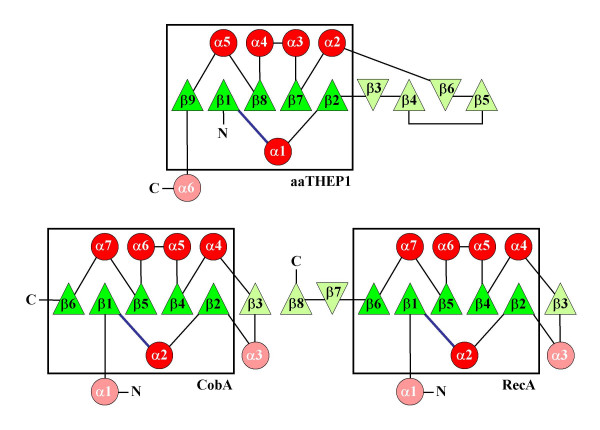
**Topology of aaTHEP1**. Topology of aaTHEP1 in comparison to RecA (pdb-code: 2REB) and Cobalamin-transferase (CobA; pdb-code: 1G5R). The location of the P-loop is indicated in blue, the common core is boxed. The drawing was prepared according to the topology as analyzed by Bauer *et al. *[7].

aaTHEP1 consists of a single compact domain confirming the gel filtration experiments as well as the resistance of aaTHEP1 to limited proteolysis [2]. It is build up of nine strands and six helixes in the sequential order βαβββββαβααβαβα (Figures 1, 2, 3) which is in agreement with previously recorded CD-spectra showing an equal ratio of β-sheets and α-helices [2]. All nine strands form a single sheet in topological order 918723465 wherein a five-stranded parallel and a four-stranded antiparallel region can be distinguished (Figure 3). Whereas the parallel part of the sheet almost lies in a plane, its antiparallel region is curved defining a convex (outer) and a concave (inner) side of the beta-structure (Figure 2). Spatially restricted to the parallel region, two α-helixes are located outside of the sheet. In contrast, a set of four helixes is distributed over the whole bended sheet at its inner side. This set consists of three parallel large α-helixes in identical N- to C-orientation who are accompanied by a further perpendicularly arranged much smaller 3/10-helix located near their N-terminal sides. The edge of the antiparallel region of the sheet forms a small bended lid that covers this smaller 3/10-helix.

In summary, the overall topology of aaTHEP1 is a central sheet with helical structures on each side. According to the CATH protein structure classification [5], aaTHEP1 is assigned to class 3.40.50.300 *i. e. *"P-loop containing nucleotide triphosphate hydrolases, homologous superfamilies with Rossmann fold topology" which are mixed alpha-beta proteins with 3-layer(α/β/α) sandwich architecture.

### Structural alignments and fold classification

For comparison with other structures in the pdb-database, the DALI algorithm was employed [6]. The closest homologue of aaTHEP1 was found to be cob(I)alamin adenosyltransferase (pdb-code: 1G5R, Z-score = 9.9) that catalyzes the final step in the conversion of vitamin B(12) to coenzyme B(12) and has a RecA-like protein fold. A comparison between the topologies of aaTHEP1, cob(I)alamin adenosyltransferase and RecA clearly shows the structural similarity (Fig. 3) despite only 9% sequence identity in the aligned region. In contrast to cob(I)alamin adenosyltransferase and RecA, aaTHEP1 contains an extension of its β-sheet consisting of strands β3-β6. We conclude that the structure of aaTHEP1 represents a variation of the RecA protein fold.

### Topology of the P-loop

**Figure 4:**
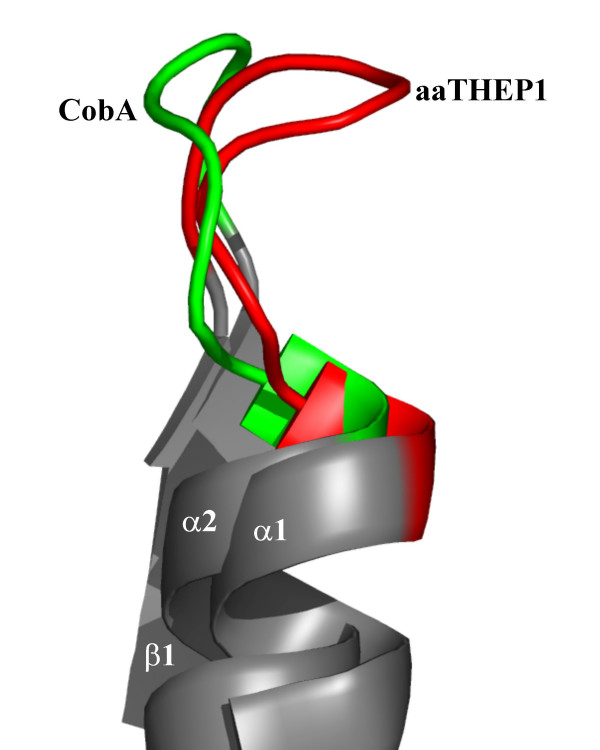
**4 P-loop topology of aaTHEP1**. Structural superposition of the P-loops of aaTHEP1 (red) and CobA (green).

Although being closest DALI-homologue, the structure of cob(I)alamin adenosyltransferase (CobA) differs significantly from aaTHEP1 within the P-loop (Figure 4). Whereas aaTHEP1 bears a P-loop typical for P-loop hydrolases, the P-loop of CobA is shorter by one amino acid which flattens its structure. This is an essential feature for the adenosyl transfer reaction [7]. Thus, we do not expect aaTHEP1 to catalyze an adenosyl transfer. A survey comparing sequences and structures of all P-loop-fold proteins led to the definition of two major divisions, the GK- and the ASCE-class of NTPases [8]. Whereas the GK-class includes all GTPases and kinases, the ASCE-class includes all further NTPases. Structurally, the GK-class enzymes contain adjacent P-loop and Walker B strands. In contrast, as it is the case for both aaTHEP1 and the RecA superfamily, the ASCE-proteins contain an additional strand between and a catalytic essential glutamate (E107 in aaTHEP1) within the Walker B motif, thus indicating that aaTHEP1 neither belongs to the group of GTPases nor to the kinase family.

### The catalytic centre

No electron density for an ADP molecule was found indicating that only the nucleotide-free protein crystallized. However, we found electron density for a phosphate ion in the putative nucleotide binding site where the β-phosphate of the nucleotide is expected. This is a usual phenomenon, since negatively charged ions are often found in empty nucleotide binding sites (*e. g. *[9]).

In other ATPases and GTPases, the aspartate residue of the consensus site DxxG (D106 in aaTHEP1) is involved in positioning a water-bridged magnesium ion presumably important for nucleotide hydrolysis [10,11]. In the nucleotide free aaTHEP1, there is also a magnesium ion at the corresponding position which is octahedrally coordinated to the hydroxyl group of T14 of the P-loop, a phosphate oxygen and four water molecules. One of these water molecules (W24) makes a hydrogen bond to D106. Thus, the arrangement of the magnesium ion is similar as this found in the nucleotide-bound conformation of other ATPases and GTPases.

**Figure 5:**
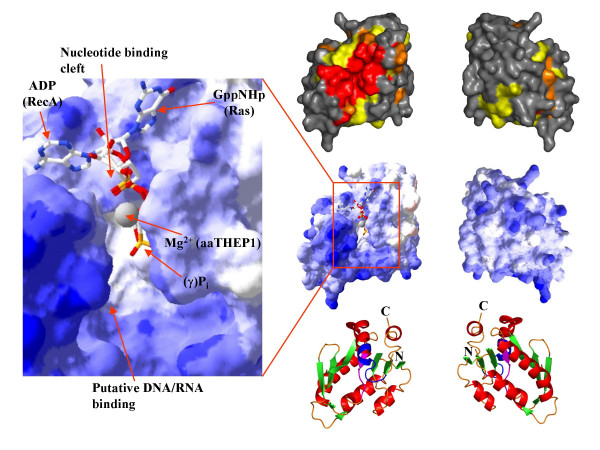
**Analysis of the protein surface of aaTHEP1**. Shown are surface and cartoon representations of aaTHEP1 in identical orientations. Conserved residues are colour coded as in Figure 1 (top). Positive electrostatic surface potentials as determined by Swiss pdb-viewer [43] are depicted blue, negative potentials in red (middle). Shown are two views related by a 180° rotation around the y-axis. In the magnified part of the active cleft, a GTP and ATP molecule can be seen. The positions of those nucleotides were constructed by superimposing the structures of the H-Ras P21 protein complexed with GppNHp (pdb-code: 5P21, [12]) and RecA complexed with ADP (pdb-code: 1MO3, [13]), respectively.

To determine possible orientations of the nucleotide which was biochemically shown to undergo hydrolysis [2], we constructed a superposition of aaTHEP1 with RAS complexed with GppNHp (pdb-code: 5P21 [12]), and RecA complexed with ADP (pdb-code: 1MO3,[13]) by aligning the P-loop including the precedent β-strand for spatial orientation (Figure 5). We then analyzed the resulting position of the nucleotides (GppNHp from Ras and ADP from RecA) relative to the aaTHEP1 surface (Figure 5). In both cases, the nucleotide would be located in a cleft of the aaTHEP1 surface and would sterically not clash with residues of aaTHEP1. The position of the phosphates is rather similar whereas the orientation of the ribose and especially the position of the base is markedly different in the ADP and GppNHp although the base would be close to conserved residues in both orientations. We cannot exactly envisage the base orientation of the nucleotide bound to aaTHEP1, but it is very likely that the overall orientation of the nucleotide and the position of the phosphates is correctly predicted. Consequently, the large remaining cleft located adjacently to the predicted position of the γ-phosphate is unoccupied. The pocket itself is rather unpolar but it is lined by a highly conserved patch of basic residues (Figure 5) to which a negatively charged cosubstrate, e. g. DNA/RNA could bind.

### The protein surface

The location of conserved residues in a protein structure often points to sites which are functionally important, e. g. the catalytic centre or conserved binding sites [14]. To detect putative binding sites of aaTHEP1, we colour coded the surface of aaTHEP1 with respect to the conservation of exposed amino acids. As can be seen in Figure 5, there is only one highly conserved region located in and around a cleft of the protein surface which includes the Walker A motif (P-loop). We conclude that this particular region represents the functionally most important site, *i. e. *the nucleotide and cosubstrate binding site of aaTHEP1. Not even a single amino acid residue conserved in all species aligned in Figure 1 can be detected on the residual protein surface of aaTHEP1. For that reason, we conclude that binding of the physiological cosubstrate is restricted to the neighbourhood of the nucleotide binding pocket.

Analysis of the electrostatic surface potential of aaTHEP1 strikingly revealed a number of positively charged clusters, whereas almost no negatively charged regions can be found (Figure 5). This is in agreement with the strong binding of aaTHEP1 to cation exchangers and its theoretical pI of 9.9. The largest positively charged spot is located in a conserved region close to the nucleotide binding cleft. Based on this observation and the similarity to the RecA protein we speculate that aaTHEP1 may be a DNA or RNA modifying enzyme. Gene functions can be predicted by searching for the conservation of operons and gene orders because genes found in gene strings, particularly in multiple genomes, can be assumed to be functionally linked [15]. For THEP1, we detected 4 genomes (*Aeropyrum pernix *K1, *Archaeoglobus fulgidus *DSM 4304, *Thermoplasma acidophilum *DSM 1728 and *Thermoplasma volcanium *GSS1) where the THEP1-gene is immediately followed by a COG1867 protein on the same strand. In *Pyrococcus furiosus*, this protein is characterized as a N2, N2-dimethylguanosine tRNA methyltransferase [16]. Thus, aaTHEP1 may also play a role in tRNA modification. Furthermore, both COG1867 proteins and THEP1 proteins can be considered to belong to the group of PACE-proteins (proteins from Archaea without assigned function that are conserved in Eukarya) [17]. PACE proteins are described being involved in fundamental cellular functions and several of them are obviously related to RNA metabolism [18].

### The human homologue

The human homologue MGC13186 (hsTHEP1) shows 39% sequence identity to aaTHEP1 (Figure 1) and was first described in a study aiming at identifying full-length ORF for all human and mouse genes[19]. No function is yet described for this protein. However, gene profiling data from UniGene are available [20]. hsTHEP1 is widely expressed in most of the examined tissues including brain, heart, lymph node, skin and pancreas whereas no expression was found in blood, thymus, bladder and spleen. It is especially highly expressed in embryonic and various tumour tissues. From these data we conclude that hsTHEP1 has a general function in many human tissues.

## Conclusion

The crystal structure of aaTHEP1 uncovered a modified RecA-like fold. Although the function of aaTHEP1 remains unclear, the structure led us to conclude that the enzyme does not belong to the group of GTPase, kinases or adenosyltransferases. Analysis of the electrostatic surface potential revealed several positively charged clusters indicating the presence of putative nucleic acid binding sites. Since aaTHEP1 has homologues in thermophilic bacteria and vertebrates it can serve as a model for the complete COG1618 protein family.

## Methods

### Nomenclature

To aid a consistent nomenclature of the THEP1 protein family we propose to adopt the name THEP1 to all members across the species, e.g. hsTHEP1 for the human protein, mmTHEP1 for the mouse protein, etc..

### Crystallization, data collection, processing, structure solution, refinement and validation

Recombinant aaTHEP1 was purified from *Escherichia coli *as described earlier [2]. Bacteria were grown in minimal media without methionine containing 50 mg/l L-selenomethionine [21]. Crystals of the dimension 250 × 80 × 35 μm^3 ^were obtained by the hanging drop method after mixing equal volumes of 13 mg/ml aaTHEP1 with reservoir buffer containing 15 % PEG-3350 and 0.1 M potassiumdihydrogenphosphate. For cryo-protection, crystals were soaked for 10 sec in 30 % PEG-3350, 200 mM potassiumdihydrogenphosphate and flash-frozen in liquid nitrogen. The diffraction data were collected at the Swiss Light Source (SLS) from a single crystal. Data were processed and scaled using XDS [22] and XSCALE [22]. The positions of the three selenium sites in the asymmetric unit were determined using SHELXD [23]. Those positions were refined and the electron density of the protein calculated by SHARP [24]. Solvent flattening and histogram matching were done by SOLOMON [25] and DM [26]. ARP/WARP was used to automatically build 85% of the backbone and sidechains [27]. For further model interpretation XFIT XtalView [28] was used. Refinements were made with Refmac [29]. PROCHECK [30] and Whatcheck [31] were used to validate the structure. Secondary structures were calculated using DSSP [32,33]. DALI-searches [6] were carried out at [34], GRATH [35] at [36] and further structural comparisons using SSAP [37] were done at [38]. BLAST was performed at [39]. Figure 1 was prepared using GeneDoc available at [40]. All figures depicting structures were prepared using PyMol [41] or Swiss pdb-viewer [42,43]. The X-Ray coordinates and structure factors have been deposited in the PDB database under pdb-code 1YE8.

## Authors' contributions

MR carried out the protein expression, purification and crystallization experiments and participated in structure determination and data analysis. OD participated in structure determination and data analysis. CK participated in data analysis. AW and MK conceived of the study, and participated in its design and coordination. MK drafted the manuscript. All authors read and approved the final manuscript.

## Figures and Tables

**Table 1:** Data collection and refinement statistics A summary of all relevant crystallographic parameters during data collection and the refinement procedures is shown.

Data collection		
Beamline	Swiss Light Source X06SA	
Space group	P2(1)	
Unit-cell dimensions	a = 35.0 Å b = 64.2 Å c = 39.6 Å	
Unit-cell angles	α = 90.0° β = 105.2° γ = 90.0°	
V_m_	2.17 Å ^3^/Da	
Solvent content	43.3 %	
Wavelength	λ_peak _= 0.97625 Å	λ_infl _= 0.97980 Å
Resolution	20 - 1.4 Å	20 - 1.4 Å
Completeness	95.3 %	95.6 %
Reflections (unique)	227088 (64230)	227487 (64213)
R_*symm*_^a,b ^total	5.7%	3.7%
R_*symm*_^a,b ^last shell	37.3%	19.2%
I/σ(I) total	11.67	19.31
I/σ(I) last shell	3.49	6.62
Wilson-B	20.86 Å^2^	19.61 Å^2^
Phasing		
Anomalous phasing power	λ_infl _= 1.7 λ_peak _= 1.8	
Anomalous phasing power last shell	λ_infl _= 0.46 λ_peak _= 0.48	
FOM	42%	
FOM last shell	15%	
FOM after solvent flattening	86%	
FOM after solvent flattening, last shell	73%	
Refinement		
Resolution	18.2 - 1.4 Å	
Reflections unique (test set)	31501(1679)	
Number of amino acids	178	
Number of atoms	1679	
Number of water molecules	293	
R_*cryst*_	16.8%	
R_*free*_	20.8%	
